# Impact of Pre-Existing Disability on Long-Term Health Care Use Following Hospitalization for COVID-19: A Population-Based Cohort Study

**DOI:** 10.1007/s11606-025-09396-8

**Published:** 2025-02-25

**Authors:** Hilary K. Brown, Thérèse A. Stukel, Hannah Chung, Samantha Lee, Yona Lunsky, Chaim M. Bell, Pavlos Bobos, Angela M. Cheung, Allan S. Detsky, Susie Goulding, Margaret Herridge, Fahad Razak, Amol A. Verma, Kieran L. Quinn

**Affiliations:** 1 Department of Health & Society, University of Toronto Scarborough, Toronto, Ontario Canada; 2 Dalla Lana School of Public Health, University of Toronto, Toronto, Ontario Canada; 3 Institute of Health Policy, Management and Evaluation, University of Toronto, Toronto, Ontario Canada; 4 ICES, Toronto, Ontario Canada; 5 Centre for Addiction, Azrieli Adult Neurodevelopmental Centre, & Mental Health, Toronto, Ontario Canada; 6 Department of Psychiatry, Temerty Faculty of Medicine, Toronto, Ontario Canada; 7 Department of Medicine, University Health Network, Toronto, Ontario Canada; 8 School of Physical Therapy, Western University, London, Ontario Canada; 9 Department of Medicine, Temerty Faculty of Medicine, Toronto, Ontario Canada; 10 Department of Medicine, Sinai Health System, Toronto, Ontario Canada; 11 COVID Long-Haulers Canada, Toronto, Canada; 12 Li Ka Shing Knowledge Institute, Unity Health Toronto, Toronto, Ontario Canada; 13 Department of Medicine, St. Michael’s Hospital, Toronto, Ontario Canada; 14 Temerty Centre for AI Research and Education in Medicine, Temerty Faculty of Medicine, University of Toronto, Toronto, Ontario Canada; 15 Temmy Latner Centre for Palliative Care, Sinai Health System, University of Toronto, 600 University Ave. 19th Floor Room 102, Toronto, Ontario M5B 1X5 Canada

**Keywords:** cohort studies, disabled persons, COVID-19, post-acute COVID-19 syndrome

## Abstract

**Background:**

Emerging evidence shows the lasting impact of SARS-CoV-2 infection on health care use and needs. Policy-makers require data on population-level service use to understand patient needs and health system impacts following hospitalization for COVID-19.

**Objective:**

To compare health service use within 12 months following hospitalization for COVID-19 among people with and without pre-existing disabilities, and to determine the extent to which such use is related to disability and other risk factors.

**Design:**

Population-based cohort study, Ontario, Canada.

**Participants:**

Adults with and without disabilities hospitalized for COVID-19, 01/25/2020–02/28/2022.

**Main Measures:**

We used Poisson regression to model adjusted rate ratios (aRR) of ambulatory care visits, diagnostic testing, emergency department (ED) visits, hospital admissions, and palliative care visits within 1-year post-discharge, comparing patients with and without disabilities. Models were adjusted sequentially for sociodemographic factors, comorbidities, and prior health service use. The importance of each set of covariates in its ability to explain observed associations was determined by calculating relative changes in disability parameter coefficients after each sequential risk-adjustment.

**Key Results:**

The cohort included 25,320 patients with disabilities and 15,953 without. In the year after hospitalization for COVID-19, people with disabilities had higher rates of ambulatory care visits, diagnostic tests, ED visits, hospital admissions, and palliative care visits. A significant proportion of these associations was explained by sociodemographic factors, comorbidities, and prior health service use. However, adjusted relative rates associated with disability remained elevated, even after adjustment, for ambulatory care visits (aRR 1.09, 95% CI 1.08, 1.10), diagnostic tests (aRR 1.14, 95% CI 1.12, 1.16), ED visits (aRR 1.25, 95% CI 1.21, 1.29), and hospital admissions (aRR 1.21, 95% CI 1.16, 1.29).

**Conclusions:**

These findings support the need to develop and evaluate models of care for the post-COVID-19 condition that address the needs of people with disabilities.

**Supplementary Information:**

The online version contains supplementary material available at 10.1007/s11606-025-09396-8.

## INTRODUCTION

The World Health Organization reported 770 million SARS-CoV-2 infections worldwide in the first 4 years of the COVID-19 pandemic.^1^ Emerging evidence shows the lasting impact of these infections on individuals’ health care use and needs, which includes development of post-COVID-19 condition.^1^ This enormous burden necessitates planning of health care resources to support people long term. To plan properly, policy-makers require data on population-level health service use to understand patient needs and associated health system impacts. It is essential that a health equity lens be applied to this planning to ensure supports are accessible to all.^2^ Yet, the needs of people with disabilities in this context have received little attention.

Globally, one in five individuals has a disability.^3,4^ Disabilities are heterogeneous and include physical, sensory, psychiatric, and developmental disabilities. People with disabilities are at heightened risk for acquiring SARS-CoV-2 infection as a result of elevated rates of immune compromise, social risk factors such as poverty, difficulty understanding and following public health guidance, and higher likelihood of living in congregate care settings where exposure to the virus is more likely.^5–8^ They are also at risk of experiencing severe short-term consequences of COVID-19 and being hospitalized due to physiological vulnerabilities related to their disability, higher rates of comorbid conditions such as cardiovascular disease (which can be exacerbated by infection), and barriers accessing high-quality health care.^5–13^ These same risk factors could place people with disabilities at risk of worse long-term outcomes after a COVID-19 hospitalization. However, the post-discharge health service use of people with disabilities, and the extent to which such use is related to their disability or other risk factors, has rarely been examined.^14^

To address these evidence gaps, we compared health services use, including ambulatory care, diagnostic tests, emergency department (ED) visits, hospitalizations, and palliative care, within 12 months following hospitalization for COVID-19 among people with and without pre-existing physical, sensory, and developmental disabilities, and investigated the extent to which such use is related to their disability or other known risk factors.

## METHODS

### Study Design and Setting

We conducted a population-based cohort study in Ontario, Canada, following the REporting of studies Conducted using Observational Routinely-collected Data (RECORD) guidelines.^15^ Ontario’s population of approximately 15 million residents receives health care services for all medically necessary care at no direct cost to residents. We accessed and analyzed data at ICES (formerly the Institute for Clinical Evaluative Sciences), an independent, non-profit research institute that uses routinely collected health administrative data derived from Ontario residents’ health care encounters for health system planning, management, and improvement.

### Data Sources

Datasets, linked at the individual level using unique encoded identifiers, included the Ontario Laboratories Information System (OLIS) and Public Health Case and Contact Management (CCM) databases for COVID-19 cases, Ontario Health Insurance Plan (OHIP) database for outpatient visits, National Ambulatory Care Reporting System for ED visits, Canadian Institute for Health Information Discharge Abstract Database for hospitalizations, Ontario Mental Health Reporting System for hospital admissions in facilities with designated mental health beds, Ontario Disability Support Program (ODSP) database for disability-related income support, COVAXON for COVID-19 vaccination receipt, Registered Persons Database for vital statistics, and Census data for socio-demographics. ICES data are valid and complete for socio-demographics, primary diagnoses for hospital stays, and physician billing claims.^16^

### Study Population

We included adults ≥ 18 years with a hospitalization for COVID-19 who were discharged alive between January 25, 2020, and February 28, 2022, excluding those with invalid age or sex data, who were ineligible for OHIP for >3 months in the year before index, or who resided in a long-term care facility. The study period was chosen as it corresponds to the first five waves of the COVID-10 pandemic, when infection rates and hospitalizations were highest.^17^ We used OLIS and CCM to identify laboratory-confirmed COVID-19 cases diagnosed within 14 days before or 3 days after admission to identify people admitted both with and for COVID-19.^18,19^

Among these patients, disability status was ascertained using algorithms developed to identify physical, sensory, and developmental disabilities in health administrative data.^20–23^ These algorithms capture diagnoses associated with functional limitations and need for accommodation when using health care,^20–24^ and have been applied in Ontario.^6,10^ A disability was deemed present if a relevant diagnosis was found in ≥ 2 physician visits (using physician billing codes), or ≥ 1 ED visits or ≥ 1 hospital admissions (using ICD-10 and DSM codes) between database inception (1988) and COVID-19 hospitalization admission date.^6,10^ In main analyses, we compared people with any disability to those without a documented disability. In pre-planned secondary analyses, we further compared (1) people with a physical disability only, sensory disability only, developmental disability only, and multiple disabilities, separately, to those without a disability, and (2) people with a disability receiving ODSP (as a proxy for greater disability-related support needs) and those not receiving ODSP, separately, to those without a disability. This latter analysis was restricted to individuals aged 18 to 64 years, the eligible age group for ODSP, and included only hospitalizations for COVID-19 up to April 30, 2021, the latest date of the ODSP database update. Finally, we undertook a sensitivity analysis excluding osteoarthritis, glaucoma, and cataracts from the disability definition—as these were the most common disability diagnoses but may vary in their impacts on functional limitations.

### Outcomes

The outcomes were the number of ambulatory care visits, diagnostic tests, ED visits not resulting in hospital admission, hospitalizations, and palliative care visits up to 12 months after the COVID-19 hospitalization discharge date. We censored at death (5.6%), 90 days after loss of OHIP eligibility (0.1%), or end of follow-up (March 31, 2022), whichever came first.

### Covariates

We measured baseline characteristics at admission: age; sex; neighborhood income quintile; Ontario Marginalization Index quintiles related to households and dwellings, material resources, age and the labor force, and racialized and newcomer populations;^25^ rurality; Comorbidities recorded in the 5 years before admission,^26^ selected based on their higher prevalence in people with disabilities^20,27,28^ and their impact on health service use; health service use in the 12 months before admission; pre-admission COVID-19 vaccination status; wave of the pandemic when the admission occurred; and, as indicators of the severity of COVID-19 illness, length of stay, diagnosis of delirium or myocardial infarction during the admission, intensive care unit admission, mechanical ventilation, Frailty Risk Score at discharge,^29^ and LACE Index score at discharge (predicting risk of death and 30-day readmission).^30^

### Analyses

We reported the baseline characteristics of patients by pre-existing disability status, and compared the balance across groups using standardized differences.^31^

We used Poisson regression to compare patients with and without disabilities on their rates of ambulatory care visits, diagnostic tests, ED visits, hospitalizations, and palliative care visits. A model offset term (in months) was used to account for differential follow-up time. Models were sequentially adjusted for sociodemographic factors, comorbidities, and prior health service use. Given their theoretical importance, all variables measured within each of these categories were included. The importance of each set of covariates in its ability to explain the association between disability status and health service use was determined by calculating the relative changes in the parameter coefficient associated with disability status after each sequential risk-adjustment model, expressed as a proportion.^30^ To do this, we used the formula: |βu − βA|/|βu| (where βu and βA are the unadjusted and adjusted parameter estimates for disability), along with bootstrapping to estimate the lower and upper boundaries of the 95% CI.^32^

In sensitivity analyses, we further adjusted for COVID-19 vaccination status, wave of the COVID-19 pandemic when the admission occurred, and severity of COVID-19 illness.

We repeated the analyses (1) for people with physical, sensory, developmental, and multiple disabilities, separately, compared to those without a disability; (2) for people with disabilities using and not using ODSP, separately, compared to those without a disability; and (3) excluding people with only osteoarthritis, glaucoma, or cataracts from the disability group.

Missing data were minimal (< 1.2%), so descriptive and unadjusted analyses included all cohort members, and multivariable modeling was performed using a complete case analysis.

Analyses used SAS version 9.4 (SAS Institute Inc., Cary, NC).

### Ethics

Data use was authorized under section 45 of Ontario’s Personal Health Information Protection Act and approved by the Mount Sinai Hospital Research Ethics Board (22–0188-E).

## RESULTS

### Baseline Characteristics

**Figure 1:**
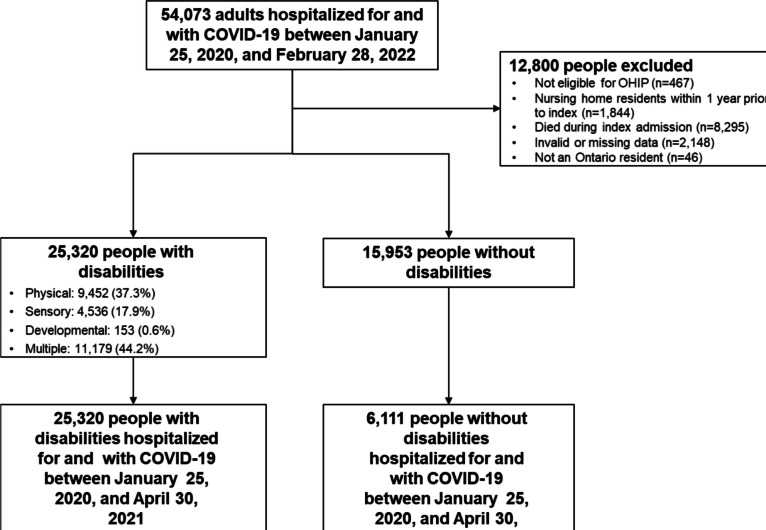
Creation of the study cohort. OHIP, Ontario Health Insurance Plan.

There were 25,320 adults with disabilities and 15,953 without disabilities hospitalized for COVID-19 between January 25, 2020, and February 28, 2022 (Fig. 1), who were followed for a median of 9.8 and 10.4 months, respectively. Compared to people without disabilities, those with disabilities were older and more likely to have multiple pre-existing comorbid conditions, higher prior health service use, prior palliative care, and COVID-19 vaccination receipt. During the index hospitalization, they were also more likely to have been diagnosed with delirium, had longer hospital length of stay, and had higher frailty and LACE scores (Tables 1 and 2).

**Table 1:** Sociodemographic Characteristics of Patients with and without Pre-existing Disabilities with a Hospitalization for COVID-19. Data Presented as *n* (%) Unless Otherwise Specified

	Disability (*N* = 25,320)	No disability (*N* = 15,953)	Standardized difference
Age, median (IQR), years	70 (58–80)	48 (35–59)	1.26
Male sex	13,144 (51.9)	8201 (51.4)	0.01
Neighborhood income quintile (Q)			
Q1 (lowest)	7897 (31.2)	4460 (28.0)	0.07
Q2	5573 (22.0)	3368 (21.1)	0.02
Q3	4629 (18.3)	3178 (19.9)	0.04
Q4	3917 (15.5)	2769 (17.4)	0.05
Q5 (highest)	3202 (12.6)	2119 (13.3)	0.02
Missing	102 (0.4)	59 (0.4)	0.01
ON-MARG: households and dwellings			
1 (least marginalized)	4965 (19.6)	3841 (24.1)	0.11
2	3702 (14.6)	2552 (16.0)	0.04
3	3804 (15.0)	2343 (14.7)	0.01
4	4828 (19.1)	2853 (17.9)	0.03
5 (most marginalized)	7760 (30.6)	4178 (26.2)	0.10
Missing	261 (1.0)	186 (1.2)	0.01
ON-MARG: material resources			
1 (least marginalized)	2992 (11.8)	1992 (12.5)	0.02
2	4210 (16.6)	2874 (18.0)	0.04
3	4892 (19.3)	3142 (19.7)	0.01
4	5456 (21.5)	3207 (20.1)	0.04
5 (most marginalized)	7509 (29.7)	4552 (28.5)	0.02
Missing	261 (1.0)	186 (1.2)	0.01
ON-MARG: age and labor force			
1 (least marginalized)	5929 (23.4)	5287 (33.1)	0.22
2	4996 (19.7)	3402 (21.3)	0.04
3	4449 (17.6)	2811 (17.6)	0.00
4	4249 (16.8)	2262 (14.2)	0.07
5 (most marginalized)	5436 (21.5)	2005 (12.6)	0.24
Missing	261 (1.0)	186 (1.2)	0.01
ON-MARG: racialized and newcomer populations			
1 (least marginalized)	2454 (9.7)	1272 (8.0)	0.06
2	3153 (12.5)	1602 (10.0)	0.08
3	4089 (16.1)	2173 (13.6)	0.07
4	5904 (23.3)	3572 (22.4)	0.02
5 (most marginalized)	9459 (37.4)	7148 (44.8)	0.15
Missing	261 (1.0)	186 (1.2)	0.01
Rural residence	1494 (5.9)	869 (5.4)	0.02
Missing	91 (0.4)	55 (0.3)	0.00

**Table 2:** Clinical Characteristics of Patients with and without Pre-existing Disabilities with a Hospitalization for COVID-19. Data Presented as *n* (%) Unless Otherwise Specified

	Disability (*N* = 25,320)	No disability (*N* = 15,953)	Standardized difference
Comorbidities	463 (1.8)	96 (0.6)	0.11
Acute myocardial infarction	1328 (5.2)	804 (5.0)	0.01
Alcohol and substance use disorder	3203 (12.7)	1172 (7.3)	0.18
Asthma	8393 (33.1)	2791 (17.5)	0.37
Cancer	2894 (11.4)	490 (3.1)	0.33
Cardiac arrhythmia	16,114 (63.6)	4704 (29.5)	0.73
Chronic hypertension	3100 (12.2)	510 (3.2)	0.34
Chronic obstructive pulmonary disease	4350 (17.2)	601 (3.8)	0.45
Congestive heart failure	4523 (17.9)	906 (5.7)	0.39
Coronary syndrome	189 (0.7)	66 (0.4)	0.04
Decompensated cirrhosis	1013 (4.0)	65 (0.4)	0.25
Deep vein thrombosis/pulmonary embolism	4295 (17.0)	1256 (7.9)	0.28
Dementia	10,951 (43.3)	3646 (22.9)	0.44
Diabetes mellitus	824 (3.3)	341 (2.1)	0.07
Immunocompromised	7293 (28.8)	3620 (22.7)	0.14
Non-psychotic disorder	2312 (9.1)	347 (2.2)	0.30
Pneumonia	639 (2.5)	303 (1.9)	0.04
Psychotic disorder	7184 (28.4)	1745 (10.9)	0.45
Renal failure	1487 (5.9)	242 (1.5)	0.23
Stroke	463 (1.8)	96 (0.6)	0.11
Health care use < 12 months before index			
Ambulatory care visits, median (IQR)	11 (6–19)	7 (3–14)	0.42
Emergency department visits, median (IQR)	0 (0–1)	0 (0–1)	0.12
Hospitalizations, median (IQR)	0 (0–1)	0 (0–0)	0.40
Palliative care	326 (1.3)	62 (0.4)	0.10
Unique prescriptions, median (IQR)	10 (1–18)	0 (0–2)	1.14
Completed primary COVID-19 vaccination series with ≥ 1 Health Canada-approved booster	2472 (9.8)	731 (4.6)	0.20
Pandemic wave			
Wave 1 (02/26/2020–08/31/2020)	1782 (7.0)	1060 (6.6)	0.02
Wave 2 (09/01/2020–02/28/2021)	5274 (20.8)	2700 (16.9)	0.10
Wave 3 (03/01/2021–07/31/2021)	7524 (29.7)	5772 (36.2)	0.14
Wave 4 (08/01/2021–12/14/2021)	1801 (7.1)	1599 (10.0)	0.10
Wave 5 (12/15/2021–02/28/2022)	8939 (35.3)	4822 (30.2)	0.11
Delirium	2914 (11.5)	708 (4.4)	0.26
Myocardial infarction at index hospitalization	255 (1.0)	146 (0.9)	0.01
ICU admission	4366 (17.2)	3042 (19.1)	0.05
Mechanical ventilation	1652 (6.5)	1154 (7.2)	0.03
Total length of stay, median (IQR), days	7 (3–13)	4 (2–9)	0.40
Hospital frailty risk score at discharge			
0	7679 (30.3)	8341 (52.3)	0.46
0.1–4.9	10,908 (43.1)	5847 (36.7)	0.13
5.0–8.9	3983 (15.7)	1097 (6.9)	0.28
≥ 9.0	2750 (10.9)	668 (4.2)	0.26
LACE score			
0–6	4789 (18.9)	6215 (39.0)	0.45
7–10	12,915 (51.0)	7693 (48.2)	0.06
≥ 11	7616 (30.1)	2045 (12.8)	0.43

### Primary Outcomes

**Figure 2:**
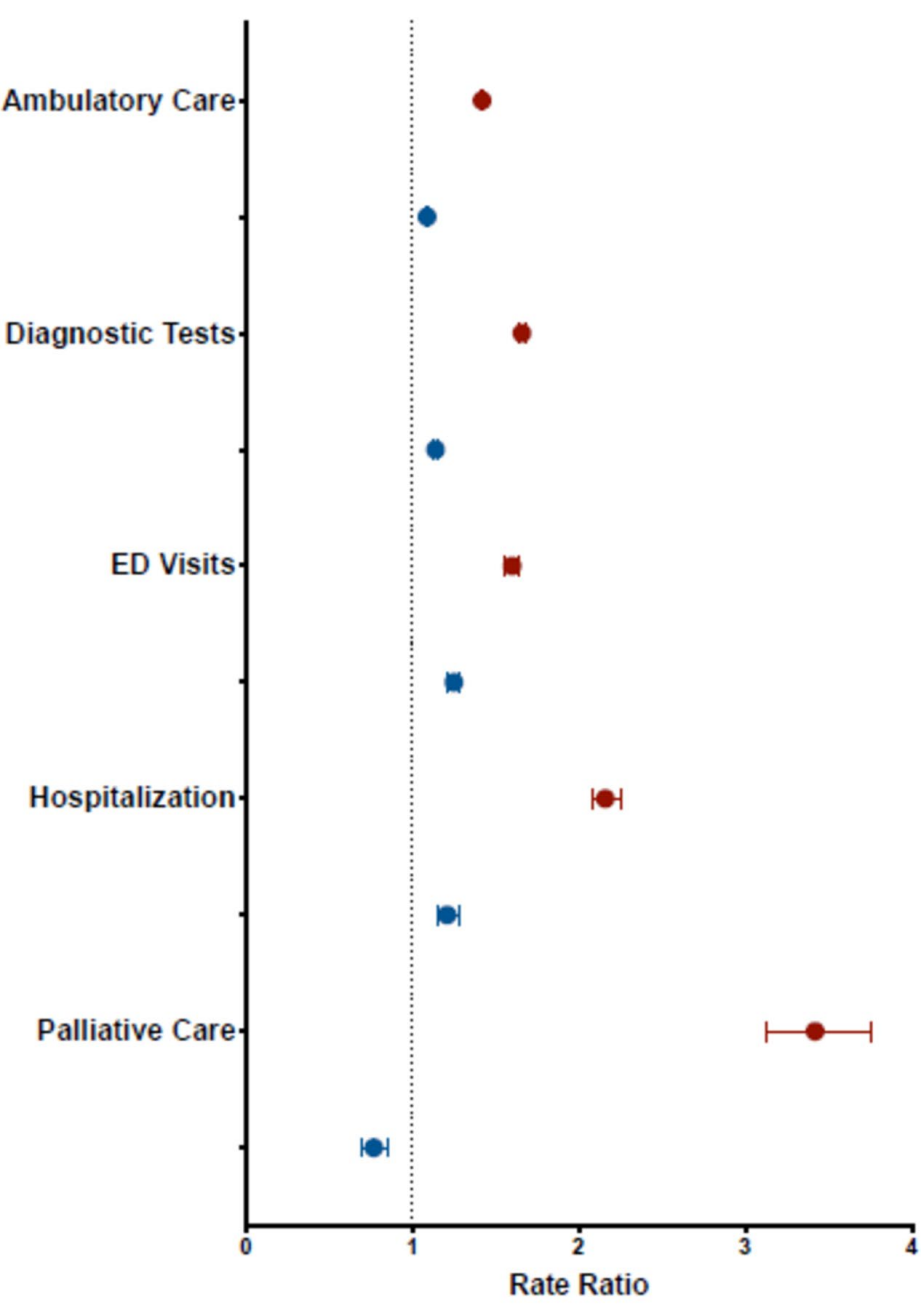
Unadjusted (red) and adjusted (blue) rate ratios of health care use among adults with and without disabilities hospitalized with COVID-19 between January 25, 2020, and February 28, 2022. Models were adjusted for a comprehensive set of sociodemographic factors, comorbidities, and prior health service use. ED, emergency department.

In the year after hospitalization for COVID-19, people with disabilities had higher rates of ambulatory care visits (1419.5 vs. 999.9 per 1000 person-months), diagnostic tests (378.9 vs. 227.8), ED visits (93.4 vs. 58.5), hospital admissions (56.2 vs. 26.0), and palliative care visits (15.2 vs. 4.4) than people without disabilities (Table 3, Fig. 2). Sociodemographic factors, comorbidities, and prior health service use explained 100.0% of the association between disability and palliative care visits and between 52.8% (hospital admissions) and 76.3% (ambulatory care visits) of the association with the other outcomes, with comorbidities being the dominant explanatory factor (Table 4). However, even after adjusting for these factors, adjusted relative rates of health service use associated with disability status remained elevated for ambulatory care visits (aRR 1.09, 95% CI 1.08, 1.10), diagnostic tests (aRR 1.14, 95% CI 1.12, 1.16), ED visits (aRR 1.25, 95% CI 1.21, 1.29), and hospital admissions (aRR 1.21, 95% CI 1.16–1.29) (Table 3; eTable 1). Findings were similar after further adjusting for COVID-19 vaccination status, pandemic wave, and severity of COVID-19 illness (eTable 2).

**Table 3:** Rates of Ambulatory Care Visits, Diagnostic Tests, Emergency Department Visits, Hospitalizations, and Palliative Care Visits, Comparing Patients with and without Pre-existing Disabilities Following Hospitalization for COVID-19

	Rate per 1000 person-months	RR (95% CI)	Adjusted RR (95% CI)*	Adjusted RR (95% CI)^†^	Adjusted RR (95% CI)^‡^
Ambulatory care visits					
Disability	1419.5	1.42 (1.41, 1.43)	1.36 (1.35, 1.37)	1.17 (1.16, 1.18)	1.09 (1.08, 1.10)
No disability	999.9	Referent	Referent	Referent	Referent
Diagnostic tests					
Disability	378.9	1.66 (1.64, 1.69)	1.49 (1.47, 1.52)	1.21 (1.19, 1.23)	1.14 (1.12, 1.16)
No disability	227.8	Referent	Referent	Referent	Referent
Emergency department visits					
Disability	93.4	1.60 (1.55, 1.64)	1.97 (1.92, 2.04)	1.42 (1.37, 1.46)	1.25 (1.21, 1.29)
No disability	58.5	Referent	Referent	Referent	Referent
Hospital admissions					
Disability	56.2	2.16 (2.08, 2.25)	1.92 (1.84, 2.00)	1.36 (1.30, 1.42)	1.21 (1.16, 1.29)
No disability	26.0	Referent	Referent	Referent	Referent
Palliative care visits					
Disability	15.2	3.42 (3.13, 3.75)	1.07 (0.97, 1.18)	0.81 (0.73, 0.90)	0.77 (0.69, 0.86)
No disability	4.4	Referent	Referent	Referent	Referent

^*^Adjusted for socio-demographics (age; sex; neighborhood income, households and dwellings, material resources, age and the labor force, and racialized and newcomer populations quintiles; rurality)

^†^Adjusted for socio-demographics and comorbidities recorded in the 5 years before the index admission

^**‡**^Adjusted for socio-demographics, comorbidities, and prior health service use (number of ambulatory care visits, emergency department visits, and hospital admissions; receipt of palliative care; and number of prescription drugs in the 12 months before the index admission)

**Table 4:** Cumulative Effects on the Disability-Health Service Use Association of Adjusting Sequentially for Blocks of Risk Factors

Outcomes	Proportion change of association that could be explained by *collective* adjustment of sequential covariate sets			Proportion change of association that could be explained by sequential covariate set adjustments	
	Socio-demographic (Model 1)	Model 1 + Comorbidities (Model 2)	Model 2 + Prior health service use (Model 3)	Comorbidities (Model 1 vs. 2)	Prior health service use (Model 2 vs. 3)
Ambulatory care visits	12.2 (8.9, 15.8)	54.8 (50.8, 59.7)	76.3 (71.6, 81.7)	48.5 (45.0, 52.9)	47.6 (40.2, 55.9)
Diagnostic tests	21.2 (16.7, 26.3)	63.2 (56.7, 71.1)	74.4 (67.5, 82.8)	53.3 (47.1, 61.5)	30.5 (23.2, 43.1)
Emergency department visits	45.5 (36.6, 56.5)	25.6 (15.5, 36.7)	52.8 (42.8, 66.7)	48.9 (42.7, 56.0)	36.5 (28.8, 51.4)
Hospital admissions	15.5 (10.6, 20.9)	60.5 (54.0, 68.2)	75.2 (68.4, 83.7)	53.2 (47.3, 60.8)	37.2 (28.3, 50.5)
Palliative care visits	94.6 (75.2, 100.0)	100.0 (93.5, 100.0)	100.0 (95.8, 100.0)	100.0 (70.0, 100.0)	25.3 (1.2, 100.0)

### Secondary Analyses

People with physical and multiple disabilities had the highest rates of ambulatory care visits and diagnostic testing, and those with developmental and multiple disabilities had the highest rates of ED visits and hospitalizations in the year after hospitalization for COVID-19 (eTable 3). Again, sociodemographic factors, comorbidities, and prior health service use were strong explanatory factors (eTable 4) but adjustment did not completely attenuate associations.

In the sub-cohort of 18- to 64-year-olds with a hospitalization before April 30, 2021, 1034 (8.8%) had disabilities and were receiving ODSP, 4603 (39.2%) had disabilities but were not receiving ODSP, and 6111 (52.0%) did not have disabilities. Disparities in comorbidities, prior health service use, and COVID-19 severity were larger for people with disabilities receiving ODSP than those not (eTable 5). People with disabilities receiving and not receiving ODSP had higher rates of health service use than those without disabilities in the year after hospitalization for COVID-19, with differences largest for people with disabilities receiving ODSP (eTable 6). Again, socio-demographics, comorbidities, and prior health service use were strong explanatory factors (eTable 7), but adjustment did not completely attenuate results.

Finally, findings were similar to the main analysis after excluding individuals with only osteoarthritis, glaucoma, or cataracts from the disability group (eTable 8).

## DISCUSSION

This large, population-based study found that in the first 2 years of the COVID-19 pandemic, people with disabilities had higher rates of health services use in the year after hospitalization for COVID-19 compared to people without disabilities, with the highest rates in people receiving disability-related income supports. While socio-demographics, comorbidities, and prior health service use were important explanatory variables, they did not completely account for observed associations. Our findings highlight the need to plan accessible and inclusive long-term health care supports for people with disabilities hospitalized for COVID-19.

Our study identified adults hospitalized for COVID-19 using established methods.^18,19^ However, findings are not generalizable to all individuals with a COVID-19 infection, only those requiring hospitalization. Given the study covered the first 2 years of the pandemic, findings also may not be generalizable to more recent years.^33^ Still, as the most severe phase of the pandemic,^17^ waves 1 to 5 have implications for planning for future public health emergencies. Ontario health records do not include self-reported disability status, so we used published algorithms that rely on medical diagnoses.^20–23^ This poses several limitations. First, we may have misclassified people with disabilities who did not have a formal diagnosis, or who did not seek health care for their disability. We also were not able to differentiate between people with disabilities who do and do not experience significant functional limitations. There was a high prevalence of people with disabilities admitted for COVID-19 in our cohort; this could be partly explained by the broad definition of disability used. However, our results were robust when analyzed by ODSP status. We lacked symptom and biochemical testing data to ascertain COVID-19 infection severity.^34^ However, we used proxies (e.g., mechanical ventilation) that likely reflect the underlying severity of COVID-19. Residual confounding could explain some of our results; for example, the age distributions of the study groups were different such that, even after adjustment, age could possibly explain some of our findings. We also could not measure broader supports available to people with disabilities (e.g., personal support workers, family). Finally, we could not determine if observed patterns are specific to COVID-19 patients, or if similar post-discharge disparities in health service use between people with and without disabilities might have been observed for other infections such as influenza.

People with disabilities are at elevated risk of acquiring SARS-CoV-2^5–8^ and having severe short-term outcomes following infection compared to those without disabilities.^5–13^ In the general population, multiple incident health conditions and symptoms such as frailty, cognitive dysfunction, fatigue, depression, anxiety, and cardiovascular sequelae are common months after hospitalization for COVID-19 and are especially elevated in those with severe infection.^19,34–36^ Mounting evidence suggests these complex symptoms associated with the post-acute phase of COVID-19 have significant impacts on health systems, in part, reflected in greater ambulatory, emergency, and inpatient health care use in the months following SARS-CoV-2 infection.^37,38^ However, few studies have examined these issues in people with disabilities. One survey of US adults found people with disabilities were more likely than those without to report symptoms >4 weeks after a COVID-19 infection and to see a health care provider, seek emergency care, or be hospitalized for these symptoms.^14^ However, surveys often exclude individuals with disabilities that impact cognition or communication,^39,40^ and the study did not ascertain whether disability preceded the COVID-19 infection. Our study used population-based data to show elevated long-term health system impacts in people with pre-existing disabilities.

Our analyses suggest these impacts are partly explained by sociodemographic, health, and health care differences between people with and without disabilities. A greater burden of comorbid conditions and older age have contributed to the vulnerability of people with disabilities to SARS-CoV-2 infection and severe short-term COVID-19-associated outcomes.^5–13^ In our cohort, adjustment for this pre-existing complexity partly, but not completely, reduced associations between disability and outpatient care, ED visits, and hospital admissions after COVID-19 hospitalization, and reversed the association for palliative care. The latter finding might signify that a higher burden of comorbidities largely drove the increased risk for palliative care in people with disabilities, but that after accounting for these factors, they have reduced access.^41,42^ Finally, incident health conditions and symptoms are elevated in patients with severe SARS-CoV-2 infection.^37,38^ Yet, while people with disabilities in our cohort had longer hospital stays, more delirium, and higher frailty and LACE scores, indicating greater severity of illness that could influence health services use, adjustment for these factors did not affect results.

Other unmeasured factors may explain the remaining risk observed among people with disabilities. For example, there may be a higher risk of unanticipated complications in this group, such as progression and worsening of symptoms related to the disability. The impact of the sequelae of COVID-19 and post-COVID-19 condition on the trajectories of disability bears further study. Observed elevated rates of ED visits and hospital admissions in patients with disabilities could also reflect known barriers to accessing high-quality outpatient care.^42,43^ While patients with disabilities in our study also tended to have higher rates of ambulatory visits and diagnostic testing, this larger volume of outpatient care use does not necessarily indicate a higher quality of care; physical, communication, and attitudinal barriers within outpatient care settings may result in residual unmet needs which are reflected in the higher emergency and inpatient care observed.^42^ Finally, findings may reflect barriers to other supports (e.g., home care, meal support, transportation) for people with disabilities, resulting in poorer long-term health.

 Our study adds to the mounting evidence of the disproportionate negative impacts of the COVID-19 pandemic on people with disabilities, showing that future emergency responses must consider their needs from the start.^2^ The higher health service use among people with disabilities in the year after hospitalization for COVID-19 shows the need for planning to ensure access to outpatient health services and reduce the need for emergency and hospital care. This presents an urgent opportunity for the development of accessible post-discharge models of care and to determine their effectiveness for people with disabilities. Several models of care have been developed to support people in the general population who experience the long-term sequelae of COVID-19, including screening and support by primary care teams, advanced testing by specialty clinics, treatment of symptoms of the post-COVID-19 condition by multidisciplinary rehabilitation services, and coordination of care referrals across multiple settings.^37^ However, care of patients following severe SARS-CoV-2 infection is fragmented and inequitable.^37^ Given the known barriers to care experienced by people with disabilities,^41–43^ there is a need to evaluate post-COVID-19 services in relation to physical accessibility of clinic spaces and transportation, efforts to address the communication needs of patients (e.g., American Sign Language interpretation), coordination of care with other medical providers managing comorbid conditions, coordination with disability-related supports (e.g., home care, transportation), as well as disability-related training needs of health care providers in these settings.^44^ Consideration of the needs of patients with disabilities following a hospitalization for COVID-19 is required in efforts to address the health system impacts of COVID-19.

## Supplementary Information

Below is the link to the electronic supplementary material.

Supplementary file1 (DOCX 61 KB)

## Data Availability

Data used for this study were housed at ICES, an independent not-for-profit corporation. While data sharing agreements prohibit ICES from making the data set publicly available, access can be granted to those who meet pre-specified criteria for confidential access, available at www.ices.on.ca/. Requests to access ICES data for research purposes may be submitted to ICES’ Data and Analytic Services. Visit http://www.ices.on.ca/DAS for more information, including contact details.
