# Supplementary material for: Impact of Pre-Existing Disability on Long-Term Health Care Use Following Hospitalization for COVID-19: A Population-Based Cohort Study
Source: J Gen Intern Med. 2025 Feb 25;40(14):3428–37. doi: 10.1007/s11606-025-09396-8 (PMC12586261; doi:10.1007/s11606-025-09396-8)
Supplement: Supplementary file 1 — Supplementary file1 (DOCX 61 KB) [file 11606_2025_9396_MOESM1_ESM.docx]

**Supplementary Information**

**Impact of Pre-Existing Disability on Long-Term Health Care Use Following Hospitalization for COVID-19**

Hilary K. Brown, PhD^1-4^; Thérèse A. Stukel, PhD^3,4^; Hannah Chung, MPH^4^; Samantha Lee, MPH^4^; Yona Lunsky, PhD CPsych^4-6^; Chaim M. Bell, MD PhD ^3,4,7^; Pavlos Bobos, PhD^8^; Angela M. Cheung, MD PhD^2,3,7,9^; Allan S. Detsky, MD PhD^3,7,9,10^; Susie Goulding^11^; Margaret Herridge, MD MSc MPH^7,9^; Fahad Razak, MD MSc^3,9,12,13^; Amol A. Verma, MD MPhil^3,9,12-14^; Kieran Quinn, MD PhD^3,4,7,9,15^

^1^Department of Health & Society, University of Toronto Scarborough, Toronto, Ontario, Canada; ^2^Dalla Lana School of Public Health, University of Toronto, Toronto, Ontario, Canada; ^3^Institute of Health Policy, Management and Evaluation, University of Toronto, Toronto, Ontario, Canada; ^4^ICES, Toronto, Ontario, Canada; ^5^Azrieli Adult Neurodevelopmental Centre, Centre for Addiction & Mental Health, Toronto, Ontario, Canada; ^6^Department of Psychiatry, Temerty Faculty of Medicine, Toronto, Ontario, Canada; ^7^Department of Medicine, University Health Network, Toronto, Ontario, Canada; ^8^School of Physical Therapy, Western University, London, Ontario, Canada; ^9^Department of Medicine, Temerty Faculty of Medicine, Toronto, Ontario, Canada; ^10^Department of Medicine, Sinai Health System, Toronto, Ontario, Canada; ^11^COVID Long-Haulers Canada; ^12^Li Ka Shing Knowledge Institute, Unity Health Toronto, Toronto, Ontario, Canada; ^13^Department of Medicine, St. Michael’s Hospital, Toronto, Ontario, Canada; ^14^Temerty Centre for AI Research and Education in Medicine, Temerty Faculty of Medicine, University of Toronto, Toronto, Ontario, Canada; ^15^Temmy Latner Centre for Palliative Care, Sinai Health System, University of Toronto, Toronto, Ontario, Canada

**eTable 1. Fit statistic for main adjusted models examining rates of ambulatory care visits, diagnostic tests, emergency department visits, hospitalizations, and palliative care visits, comparing patients with and without pre-existing disabilities following hospitalization for COVID-19.**

**eTable 2. Rates of ambulatory care visits, diagnostic tests, emergency department visits, hospitalizations, and palliative care visits, comparing patients with and without pre-existing disabilities following hospitalization for COVID-19, further adjusted for COVID-19 vaccination status, pandemic wave, and severity of COVID-19 illness.**

**eTable 3.** **Rates of ambulatory care visits, diagnostic tests, emergency department visits, hospitalizations, and palliative care visits, comparing patients with and without pre-existing disabilities following hospitalization for COVID-19, by disability type.**

**eTable 4.** **Cumulative effects on the disability-health service use association of adjusting sequentially for blocks of risk factors, by disability type.**

**eTable 5.** **Baseline characteristics of patients with and without pre-existing disabilities with a hospitalization for COVID-19, by Ontario Disability Support Program (ODSP) status.**

**eTable 6.** **Rates of ambulatory care visits, diagnostic tests, emergency department visits, hospitalizations, and palliative care visits, comparing patients with and without pre-existing disabilities following hospitalization for COVID-19, by Ontario Disability Support Program status.**

**eTable 7.** **Cumulative effects on the disability-health service use association of adjusting sequentially for blocks of risk factors, by Ontario Disability Support Program status.**

**eTable 8.** **Rates of ambulatory care visits, diagnostic tests, emergency department visits, hospitalizations, and palliative care visits, comparing patients with and without pre-existing disabilities following hospitalization for COVID-19, using the alternative definition of disability**.**

**eTable 1. Fit statistic for main adjusted models examining rates of ambulatory care visits, diagnostic tests, emergency department visits, hospitalizations, and palliative care visits, comparing patients with and without pre-existing disabilities following hospitalization for COVID-19.**

| **Outcome** | **Deviance** | **Scaled Deviance** | **Pearson Chi-Square** | **Scaled Pearson X^2^** | **Log Likelihood** | **Full Log Likelihood** | **AIC** | **AICC** | **BIC** |
| --- | --- | --- | --- | --- | --- | --- | --- | --- | --- |
| **Ambulatory care visits** |  |  |  |  |  |  |  |  |  |
| DF | 4.10E+04 | 4.10E+04 | 4.10E+04 | 4.10E+04 |  |  |  |  |  |
| Value | 191011.5 | 191011.5 | 225861.7 | 225861.7 | 591282.7 | -166817 | 333752.4 | 333752.6 | 334261.5 |
| Value/DF | 4.6346 | 4.6346 | 5.4802 | 5.4802 |  |  |  |  |  |
| **Diagnostic tests** |  |  |  |  |  |  |  |  |  |
| DF | 4.10E+04 | 4.10E+04 | 4.10E+04 | 4.10E+04 |  |  |  |  |  |
| Value | 185060.1 | 185060.1 | 266125.9 | 266125.9 | 17232.45 | -122303 | 244723.4 | 244723.6 | 245232.5 |
| Value/DF | 4.4902 | 4.4902 | 6.4572 | 6.4572 |  |  |  |  |  |
| **Emergency department visits** |  |  |  |  |  |  |  |  |  |
| DF | 4.10E+04 | 4.10E+04 | 4.10E+04 | 4.10E+04 |  |  |  |  |  |
| Value | 61207.61 | 61207.61 | 108896.3 | 108896.3 | -23633.7 | -44416.8 | 88951.58 | 88951.75 | 89460.63 |
| Value/DF | 1.4851 | 1.4851 | 2.6422 | 2.6422 |  |  |  |  |  |
| **Hospital admissions** |  |  |  |  |  |  |  |  |  |
| DF | 4.10E+04 | 4.10E+04 | 4.10E+04 | 4.10E+04 |  |  |  |  |  |
| Value | 40839.18 | 40839.18 | 85136.6 | 85136.6 | -25015.6 | -30366 | 60850 | 60850.17 | 61359.05 |
| Value/DF | 0.9909 | 0.9909 | 2.0657 | 2.0657 |  |  |  |  |  |
| **Palliative care** |  |  |  |  |  |  |  |  |  |
| DF | 4.10E+04 | 4.10E+04 | 4.10E+04 | 4.10E+04 |  |  |  |  |  |
| Value | 21317.09 | 21317.09 | 364465.4 | 364465.4 | -8184.14 | -12002.8 | 24123.51 | 24123.69 | 24632.56 |
| Value/DF | 0.5172 | 0.5172 | 8.8432 | 8.8432 |  |  |  |  |  |

**eTable 2. Rates of ambulatory care visits, diagnostic tests, emergency department visits, hospitalizations, and palliative care visits, comparing patients with and without pre-existing disabilities following hospitalization for COVID-19, further adjusted for COVID-19 vaccination status, pandemic wave, and severity of COVID-19 illness.**

|  | **Rate per 1000 person-months** | **Adjusted RR**  **(95% CI)** * | **Adjusted RR**  **(95% CI)** † | **Adjusted RR**  **(95% CI)** **‡** |
| --- | --- | --- | --- | --- |
| **Ambulatory care visits** |  |  |  |  |
| No disability | 1419.5 | 1.09 (1.08, 1.10) | 1.09 (1.08, 1.10) | 1.08 (1.07, 1.09) |
| Disability | 999.9 | Referent | Referent | Referent |
| **Diagnostic tests** |  |  |  |  |
| No disability | 378.9 | 1.14 (1.12, 1.18) | 1.14 (1.12, 1.16) | 1.13 (1.11, 1.15) |
| Disability | 227.8 | Referent | Referent | Referent |
| **Emergency department visits** |  |  |  |  |
| No disability | 93.4 | 1.25 (1.21, 1.29) | 1.25 (1.21, 1.29) | 1.22 (1.18, 1.26) |
| Disability | 58.5 | Referent | Referent | Referent |
| **Hospital admissions** |  |  |  |  |
| No disability | 56.2 | 1.21 (1.15, 1.26) | 1.21 (1.16, 1.27) | 1.19 (1.13, 1.25) |
| Disability | 26.0 | Referent | Referent | Referent |
| **Palliative care visits** |  |  |  |  |
| No disability | 15.2 | 0.77 (0.69, 0.86) | 0.79 (0.71, 0.87) | 0.83 (0.74, 0.92) |
| Disability | 4.4 | Referent | Referent | Referent |

* Adjusts for socio-demographics, comorbidities, prior health service use, and COVID-19 vaccination status.

† Adjusts for socio-demographics, comorbidities, prior health service use, COVID-19 vaccination status, and pandemic wave.

**‡** Adjusts for socio-demographics, comorbidities, prior health service use, COVID-19 vaccination status, pandemic wave, and severity of COVID-19 illness in hospital (delirium, myocardial infarction at index hospitalization, intensive care unit admission, mechanical ventilation, total length of stay, Frailty Risk Score at discharge, and LACE Index score at discharge).

**eTable 3. Rates of ambulatory care visits, diagnostic tests, emergency department visits, hospitalizations, and palliative care visits, comparing patients with and without pre-existing disabilities following hospitalization for COVID-19, by disability type.**

|  | **Rate per 1000 person-months** | **RR**  **(95% CI)** | **Adjusted RR**  **(95% CI)** * | **Adjusted RR**  **(95% CI)** † | **Adjusted RR**  **(95% CI)** **‡** |
| --- | --- | --- | --- | --- | --- |
| **Ambulatory care visits** |  |  |  |  |  |
| Physical disability | 1337.5 | 1.34 (1.33, 1.35) | 1.33 (1.32, 1.35) | 1.17 (1.16, 1.18) | 1.10 (1.09, 1.11) |
| Sensory disability | 1301.4 | 1.30 (1.29, 1.32) | 1.27 (1.25, 1.28) | 1.14 (1.13, 1.16) | 1.09 (1.07, 1.10) |
| Intellectual/developmental disability | 1068.5 | 1.07 (1.01, 1.13) | 1.11 (1.05, 1.17) | 1.02 (0.96, 1.07) | 0.83 (0.78, 0.87) |
| Multiple disabilities | 1551.9 | 1.55 (1.54, 1.56) | 1.50 (1.48, 1.51) | 1.21 (1.20, 1.22) | 1.07 (1.06, 1.08) |
| No disability | 999.9 | Referent | Referent | Referent | Referent |
|  |  |  |  |  |  |
| **Diagnostic tests** |  |  |  |  |  |
| Physical disability | 366.9 | 1.61 (1.58, 1.64) | 1.51 (1.49, 1.54) | 1.26 (1.24, 1.28) | 1.19 (1.17, 1.21) |
| Sensory disability | 344.4 | 1.51 (1.48, 1.54) | 1.37 (1.34, 1.40) | 1.17 (1.14, 1.20) | 1.13 (1.11, 1.16) |
| Intellectual/developmental disability | 203.4 | 0.89 (0.79, 1.01) | 0.87 (0.77, 0.99) | 0.79 (0.70, 0.90) | 0.78 (0.69, 0.88) |
| Multiple disabilities | 407.7 | 1.79 (1.76, 1.82) | 1.57 (1.54, 1.60) | 1.15 (1.13, 1.18) | 1.05 (1.03, 1.07) |
| No disability | 227.8 | Referent | Referent | Referent | Referent |
|  |  |  |  |  |  |
| **Emergency department visits** |  |  |  |  |  |
| Physical disability | 101.4 | 1.73 (1.67, 1.79) | 1.94 (1.87, 2.00) | 1.40 (1.35, 1.45) | 1.27 (1.22, 1.31) |
| Sensory disability | 58.0 | 0.99 (0.94, 1.04) | 1.34 (1.28, 1.41) | 1.13 (1.07, 1.19) | 1.06 (1.01, 1.12) |
| Intellectual/developmental disability | 198.5 | 3.39 (2.98, 3.86) | 2.57 (2.26, 2.92) | 1.59 (1.39, 1.81) | 1.52 (1.33, 1.74) |
| Multiple disabilities | 100.6 | 1.72 (1.66, 1.77) | 2.51 (2.41, 2.60) | 1.65 (1.59, 1.72) | 1.32 (1.26, 1.37) |
| No disability | 58.3 | Referent | Referent | Referent | Referent |
|  |  |  |  |  |  |
| **Hospital admissions** |  |  |  |  |  |
| Physical disability | 49.4 | 1.90 (1.81, 1.99) | 1.79 (1.70, 1.88) | 1.32 (1.25, 1.38) | 1.19 (1.13, 1.26) |
| Sensory disability | 42.0 | 1.61 (1.52, 1.72) | 1.57 (1.47, 1.68) | 1.23 (1.15, 1.31) | 1.16 (1.09, 1.24) |
| Intellectual/developmental disability | 65.3 | 2.51 (2.01, 3.14) | 2.19 (1.75, 2.74) | 1.61 (1.28, 2.01) | 1.56 (1.24, 1.95) |
| Multiple disabilities | 68.6 | 2.64 (2.53, 2.75) | 2.43 (2.30, 2.56) | 1.51 (1.43, 1.59) | 1.26 (1.19, 1.33) |
| No disability | 26.0 | Referent | Referent | Referent | Referent |
|  |  |  |  |  |  |
| **Palliative care visits** |  |  |  |  |  |
| Physical disability | 10.0 | 2.26 (2.02, 2.52) | 1.21 (1.08, 1.35) | 0.98 (0.87, 1.09) | 0.97 (0.87, 1.09) |
| Sensory disability | 10.5 | 2.36 (2.07, 2.69) | 0.78 (0.68, 0.89) | 0.67 (0.58, 0.77) | 0.59 (0.51, 0.68) |
| Intellectual/developmental disability | 0.8-4.1 | 0.37 (0.09, 1.49) | 0.50 (0.13, 2.02) | 0.62 (0.15, 2.48) | 0.29 (0.07, 1.16) |
| Multiple disabilities | 22.2 | 5.00 (4.55, 5.50) | 1.09 (0.97, 1.21) | 0.73 (0.65, 0.82) | 0.68 (0.60, 0.76) |
| No disability | 4.4 | Referent | Referent | Referent | Referent |

* Adjusted for socio-demographics (age; sex; neighbourhood income, households and dwellings, material resources, age and the labour force, and racialized and newcomer populations quintiles; rurality).

† Adjusts for socio-demographics and comorbidities recorded in the 5 years before the index admission.

‡ Adjusts for socio-demographics, comorbidities, and prior health service use (number of ambulatory care visits, emergency department visits, and hospital admissions; receipt of palliative care; and number of prescription drugs in the 12 months before the index admission.

**eTable 4. Cumulative effects on the disability-health service use association of adjusting sequentially for blocks of risk factors, by disability type.**

| **Outcomes** | **Disability status** | **Proportion change of association that could be explained by collective adjustment of sequential covariate sets** | | | **Proportion change of association that could be explained by sequential covariate set adjustments** | |
| --- | --- | --- | --- | --- | --- | --- |
|  |  | **Sociodemographic  (Model 1)** | **Model 1 + Comorbidities  (Model 2)** | **Model 2 + Prior health service use  (Model 3)** | **Comorbidities  (Model 1 vs. 2)** | **Prior health**  **service use (Model 2 vs. 3)** |
| Ambulatory  care visits | Physical disability | 1.2 (0.1, 4.0) | 46.7 (42.0, 52.4) | 66.8 (61.2, 73.9) | 46.1 (42.0, 51.2) | 37.8 (30.6, 47.1) |
|  | Sensory disability | 9.8 (5.0, 14.7) | 49.7 (42.7, 57.6) | 68.6 (60.4, 78.2) | 44.2 (38.2, 51.9) | 37.6 (27.4, 51.5) |
|  | IDD | 58.2 (10.7, 100.0) | 78.3 (4.7, 100.0) | 100.0 (8.1, 100.0) | 86.3 (24.0, 100.0) | 100.0 (5.7, 100.0) |
|  | Multiple disabilities | 8.5 (4.4, 12.5) | 56.7 (51.9, 61.7) | 84.7 (78.9, 91.2) | 52.6 (48.8, 57.3) | 64.8 (53.9, 77.9) |
| Diagnostic  tests | Physical disability | 13.0 (9.7, 16.7) | 51.7 (45.6, 59.2) | 63.0 (56.2, 71.3) | 44.5 (39.0, 51.8) | 23.4 (17.0, 32.6) |
|  | Sensory disability | 24.0 (17.3, 31.8) | 62.4 (52.1, 75.5) | 70.0 (59.6, 83.8) | 50.5 (40.5, 64.7) | 20.1 (11.3, 38.4) |
|  | IDD | 21.5 (0.8, 100.0) | 100.0 (12.5, 100.0) | 100.0 (5.6, 100.0) | 72.4 (6.8, 100.0) | 5.9 (0.8, 100.0) |
|  | Multiple disabilities | 22.1 (15.9, 28.4) | 75.7 (67.7, 85.4) | 91.2 (82.0, 100.0) | 68.8 (60.4, 79.8) | 64.0 (43.5, 100.0) |
| Emergency department visits | Physical disability | 20.8 (14.6, 28.0) | 38.8 (29.6, 51.0) | 56.7 (48.0, 71.0) | 49.4 (41.6, 59.4) | 29.2 (16.1, 47.4) |
|  | Sensory disability | 100.0 (100.0, 100.0) | 100.0 (100.0, 100.0) | 100.0 (22.2, 100.0) | 59.5 (43.3, 93.2) | 51.6 (25.3, 100.0) |
|  | IDD | 22.7 (15.4, 37.8) | 62.2 (43.9, 100.0) | 65.8 (40.2, 97.7) | 51.1 (30.5, 100.0) | 9.5 (1.8, 100.0) |
|  | Multiple disabilities | 69.8 (56.8, 83.6) | 7.2 (0.4, 20.7) | 49.1 (34.2, 71.4) | 45.3 (40.1, 52.2) | 45.2 (33.5, 66.1) |
| Hospital  admissions | Physical disability | 9.4 (5.5, 13.9) | 57.2 (49.6, 66.4) | 72.4 (63.6, 83.0) | 52.8 (45.6, 62.0) | 35.6 (23.8, 53.0) |
|  | Sensory disability | 5.5 (0.3, 15.2) | 56.6 (43.5, 72.6) | 68.7 (54.7, 86.5) | 54.1 (42.5, 69.1) | 27.8 (13.9, 52.3) |
|  | IDD | 14.7 (8.7, 24.3) | 48.5 (32.7, 74.2) | 52.0 (33.4, 79.7) | 39.6 (22.2, 67.9) | 6.6 (0.5, 39.2) |
|  | Multiple disabilities | 8.5 (2.8, 14.7) | 57.9 (51.1, 64.9) | 76.0 (68.0, 83.6) | 53.9 (48.4, 60.2) | 43.0 (33.0, 54.8) |
| Palliative  care visits | Physical disability | 76.8 (53.6, 100.0) | 100.0 (73.3, 100.0) | 100.0 (73.0, 100.0) | 100.0 (40.4, 100.0) | 19.7 (1.5, 100.0) |
|  | Sensory disability | 100.0 (88.7, 100.0) | 100.0 (100.0, 100.0) | 100.0 (100.0, 100.0) | 62.3 (15.3, 100.0) | 29.6 (1.2, 100.0) |
|  | IDD | 30.4 (1.8, 100.0) | 50.9 (1.0, 100.0) | 26.5 (0.9, 100.0) | 29.4 (0.3, 100.0) | 100.0 (0.2, 100.0) |
|  | Multiple disabilities | 94.9 (77.8, 100.0) | 100.0 (98.0, 100.0) | 100.0 (100.0, 100.0) | 100.0 (89.2, 100.0) | 25.2 (1.5, 100.0) |

Abbreviations: IDD = intellectual and developmental disabilities.

**eTable 5. Baseline characteristics of patients with and without pre-existing disabilities with a hospitalization for COVID-19, by Ontario Disability Support Program (ODSP) status.** Data presented as n (%) unless otherwise specified.

|  | **Disability**  **– ODSP**  **(N = 1,034)** | **Standardized difference vs. no disability** | **Disability**  **– No ODSP**  **(N = 3,603)** | **Standardized difference vs. no disability** | **No disability**  **(N = 6,111)** |
| --- | --- | --- | --- | --- | --- |
| **Sociodemographic characteristics** |  |  |  |  |  |
| Age, median (IQR) years | 55 (44-60) | 0.47 | 56 (49-60) | 0.66 | 48 (37-56) |
| Male sex | 531 (51.4) | 0.09 | 2,015 (55.9) | 0.00 | 3,415 (55.9) |
| Neighbourhood income quintile (Q) |  |  |  |  |  |
| Q1 (lowest) | 493 (47.7) | 0.41 | 889 (24.7) | 0.08 | 1,729 (28.3) |
| Q2 | 216 (20.9) | 0.01 | 760 (21.1) | 0.00 | 1,296 (21.2) |
| Q3 | 152 (14.7) | 0.14 | 745 (20.7) | 0.02 | 1,210 (19.8) |
| Q4 | 105 (10.2) | 0.20 | 661 (18.3) | 0.03 | 1,040 (17.0) |
| Q5 (highest) | 60-64 | 0.24 | 529 (14.7) | 0.04 | 811 (13.3) |
| Missing | < 6 | 0.02 | 19 (0.5) | 0.02 | 25 (0.4) |
| ONMARG: Households and dwellings |  |  |  |  |  |
| 1 (least marginalized) | 141 (13.6) | 0.36 | 1,063 (29.5) | 0.03 | 1,709 (28.0) |
| 2 | 102 (9.9) | 0.16 | 596 (16.5) | 0.04 | 925 (15.1) |
| 3 | 128 (12.4) | 0.03 | 502 (13.9) | 0.02 | 816 (13.4) |
| 4 | 197 (19.1) | 0.05 | 612 (17.0) | 0 | 1,038 (17.0) |
| 5 (most marginalized) | 453 (43.8) | 0.38 | 792 (22.0) | 0.09 | 1,577 (25.8) |
| Missing | 13 (1.3) | 0.05 | 38 (1.1) | 0.03 | 46 (0.8) |
| ONMARG: Material resources |  |  |  |  |  |
| 1 (least marginalized) | 71 (6.9) | 0.17 | 437 (12.1) | 0.01 | 729 (11.9) |
| 2 | 100 (9.7) | 0.24 | 647 (18.0) | 0 | 1,089 (17.8) |
| 3 | 148 (14.3) | 0.16 | 798 (22.1) | 0.05 | 1,238 (20.3) |
| 4 | 211 (20.4) | 0.00 | 758 (21.0) | 0.02 | 1,238 (20.3) |
| 5 (most marginalized) | 491 (47.5) | 0.39 | 925 (25.7) | 0.07 | 1,771 (29.0) |
| Missing | 13 (1.3) | 0.05 | 38 (1.1) | 0.03 | 46 (0.8) |
| ONMARG: Age and labour force |  |  |  |  |  |
| 1 (least marginalized) | 306 (29.6) | 0.15 | 1,159 (32.2) | 0.1 | 2,243 (36.7) |
| 2 | 258 (25.0) | 0.06 | 845 (23.5) | 0.02 | 1,378 (22.5) |
| 3 | 159 (15.4) | 0.04 | 639 (17.7) | 0.02 | 1,035 (16.9) |
| 4 | 159 (15.4) | 0.07 | 511 (14.2) | 0.03 | 795 (13.0) |
| 5 (most marginalized) | 139 (13.4) | 0.11 | 411 (11.4) | 0.04 | 614 (10.0) |
| Missing | 13 (1.3) | 0.05 | 38 (1.1) | 0.03 | 46 (0.8) |
| ONMARG: Racialized and newcomer populations |  |  |  |  |  |
| 1 (least marginalized) | 61 (5.9) | 0.09 | 209 (5.8) | 0.08 | 246 (4.0) |
| 2 | 74 (7.2) | 0.03 | 309 (8.6) | 0.08 | 398 (6.5) |
| 3 | 166 (16.1) | 0.13 | 423 (11.7) | 0.00 | 710 (11.6) |
| 4 | 206 (19.9) | 0.07 | 826 (22.9) | 0.00 | 1,403 (23.0) |
| 5 (most marginalized) | 514 (49.7) | 0.09 | 1,798 (49.9) | 0.08 | 3,308 (54.1) |
| Missing | 13 (1.3) | 0.05 | 38 (1.1) | 0.03 | 46 (0.8) |
| Rural residence | 41 (4.0) | 0.08 | 124 (3.4) | 0.05 | 154 (2.5) |
| Missing | < 6 | 0.02 | 17 (0.5) | 0.01 | 24 (0.4) |
| **Pre-existing health and health service use** |  |  |  |  |  |
| Comorbidities |  |  |  |  |  |
| Acute myocardial infarction | 9 (0.9) | 0.07 | 42 (1.2) | 0.10 | 19 (0.3) |
| Alcohol and substance use disorder | 179 (17.3) | 0.44 | 170 (4.7) | 0.04 | 244 (4.0) |
| Asthma | 183 (17.7) | 0.26 | 496 (13.8) | 0.15 | 549 (9.0) |
| Cancer | 233 (22.5) | 0.19 | 887 (24.6) | 0.24 | 932 (15.3) |
| Cardiac arrhythmia | 46 (4.4) | 0.14 | 149 (4.1) | 0.13 | 121 (2.0) |
| Chronic hypertension | 508 (49.1) | 0.41 | 1,596 (44.3) | 0.31 | 1,793 (29.3) |
| Chronic obstructive pulmonary disease | 95 (9.2) | 0.34 | 128 (3.6) | 0.12 | 102 (1.7) |
| Congestive heart failure | 118 (11.4) | 0.37 | 186 (5.2) | 0.16 | 132 (2.2) |
| Coronary syndrome | 94 (9.1) | 0.23 | 284 (7.9) | 0.18 | 221 (3.6) |
| Decompensated cirrhosis | 17 (1.6) | 0.14 | 24 (0.7) | 0.06 | 16 (0.3) |
| Deep vein thrombosis/pulmonary embolism | 48 (4.6) | 0.17 | 100 (2.8) | 0.07 | 106 (1.7) |
| Dementia | < 6 | 0.08 | < 6 | 0.03 | < 6 |
| Diabetes mellitus | 479 (46.3) | 0.48 | 1,285 (35.7) | 0.26 | 1,469 (24.0) |
| Immunocompromised | 217 (21.0) | 0.46 | 304 (8.4) | 0.11 | 349 (5.7) |
| Non-psychotic disorder | 518 (50.1) | 0.62 | 1,085 (30.1) | 0.20 | 1,314 (21.5) |
| Pneumonia | 167 (16.2) | 0.54 | 119 (3.3) | 0.13 | 82 (1.3) |
| Psychotic disorder | 124 (12.0) | 0.41 | 50 (1.4) | 0.03 | 106 (1.7) |
| Renal failure | 295 (28.5) | 0.51 | 518 (14.4) | 0.16 | 567 (9.3) |
| Stroke | 47 (4.5) | 0.22 | 88 (2.4) | 0.11 | 62 (1.0) |
| Health service use < 12 months before index |  |  |  |  |  |
| Ambulatory care visits, median (IQR) | 13 (7-23) | 0.65 | 10 (5-17) | 0.35 | 7 (3-13) |
| Emergency department visits, median (IQR) | 1 (0-3) | 0.44 | 0 (0-1) | 0.10 | 0 (0-1) |
| Hospitalizations, median (IQR) | 0 (0-1) | 0.71 | 0 (0-0) | 0.18 | 0 (0-0) |
| Palliative care | < 6 | 0.06 | 6 (0.2) | 0.01 | 8 (0.1) |
| Unique prescriptions, median (IQR) | 14 (8-22) | 2.36 | 0 (0-1) | 0.14 | 0 (0-1) |
| COVID-19 vaccination status |  |  |  |  |  |
| Unvaccinated | 959 (92.7) | 0.13 | 3,399 (94.3) | 0.07 | 5,855 (95.8) |
| Initiated primary series | 67 (6.5) | 0.12 | 186 (5.2) | 0.06 | 236 (3.9) |
| Completed primary series | 8 (0.8) | 0.06 | 18 (0.5) | 0.03 | 15-19 |
| Completed primary series with ≥ 1 HC booster | 0 (0.0) | 0.02 | 0 (0.0) | 0.02 | < 6 |
| Pandemic wave |  |  |  |  |  |
| Wave 1 (02/26/2020-08/31/2020) | 149 (14.4) | 0.01 | 590 (16.4) | 0.05 | 896 (14.7) |
| Wave 2 (09/01/2020-02/28/2021) | 421 (40.7) | 0.1 | 1,323 (36.7) | 0.01 | 2,204 (36.1) |
| Wave 3 (03/01/2021-07/31/2021) | 459-463 (44.6) | 0.09 | 1,678 (46.6) | 0.05 | 2,996 (49.0) |
| Wave 4 (08/01/2021-12/14/2021) | < 6 | 0.02 | 12 (0.3) | 0.02 | 10-14 |
| Wave 5 (12/15/2021-02/28/2022) | < 6 | 0.03 | 0 (0.0) | 0.03 | < 6 |
| **Index hospitalization characteristics** |  |  |  |  |  |
| Delirium | 85 (8.2) | 0.16 | 189 (5.2) | 0.04 | 267 (4.4) |
| Myocardial infarction at index hospitalization | 0 (0.0) | 0.1 | 31 (0.9) | 0.05 | 30 (0.5) |
| ICU admission | 237 (22.9) | 0.02 | 896 (24.9) | 0.07 | 1,338 (21.9) |
| Mechanical ventilation | 122 (11.8) | 0.06 | 420 (11.7) | 0.06 | 599 (9.8) |
| Total length of stay, median (IQR), days | 7 (3-15) | 0.29 | 6 (3-11) | 0.21 | 5 (3-9) |
| Hospital frailty risk score at discharge |  |  |  |  |  |
| 0 | 305 (29.5) | 0.44 | 1,480 (41.1) | 0.2 | 3,103 (50.8) |
| 0.1-4.9 | 489 (47.3) | 0.17 | 1,572 (43.6) | 0.1 | 2,379 (38.9) |
| 5.0-8.9 | 151 (14.6) | 0.28 | 330 (9.2) | 0.12 | 371 (6.1) |
| ≥ 9.0 | 89 (8.6) | 0.18 | 221 (6.1) | 0.09 | 258 (4.2) |
| LACE score |  |  |  |  |  |
| 0-6 | 185 (17.9) | 0.42 | 935 (26.0) | 0.22 | 2,203 (36.0) |
| 7-10 | 502 (48.5) | 0.08 | 2,001 (55.5) | 0.06 | 3,210 (52.5) |
| ≥ 11 | 347 (33.6) | 0.55 | 667 (18.5) | 0.2 | 698 (11.4) |

**eTable 6. Rates of ambulatory care visits, diagnostic tests, emergency department visits, hospitalizations, and palliative care visits, comparing patients with and without pre-existing disabilities following hospitalization for COVID-19, by Ontario Disability Support Program status.**

|  | **Rate per 1000 person-months** | **RR**  **(95% CI)** | **Adjusted RR**  **(95% CI)** * | **Adjusted RR**  **(95% CI)** † | **Adjusted RR**  **(95% CI)** **‡** |
| --- | --- | --- | --- | --- | --- |
| **Ambulatory care visits** |  |  |  |  |  |
| Disability and ODSP | 1552.0 | 1.64 (1.61, 1.66) | 1.59 (1.56, 1.61) | 1.16 (1.14, 1.18) | 1.07 (1.05, 1.09) |
| Disability and no ODSP | 1321.4 | 1.39 (1.38, 1.41) | 1.34 (1.32, 1.36) | 1.20 (1.18, 1.21) | 1.11 (1.09, 1.12) |
| No disability | 949.0 | Referent | Referent | Referent | Referent |
|  |  |  |  |  |  |
| **Diagnostic tests** |  |  |  |  |  |
| Disability and ODSP | 463.2 | 2.31 (2.24, 2.39) | 2.05 (1.98, 2.12) | 1.20 (1.19, 1.24) | 1.08 (1.04, 1.12) |
| Disability and no ODSP | 326.8 | 1.63 (1.59, 1.67) | 1.44 (1.41, 1.48) | 1.23 (1.20, 1.26) | 1.18 (1.15, 1.21) |
| No disability | 200.3 | Referent | Referent | Referent | Referent |
|  |  |  |  |  |  |
| **Emergency department visits** |  |  |  |  |  |
| Disability and ODSP | 239.7 | 4.70 (4.48, 4.94) | 4.29 (4.07, 4.52) | 2.00 (1.89, 2.12) | 1.63 (1.53, 1.74) |
| Disability and no ODSP | 73.6 | 1.44 (1.38, 1.52) | 1.68 (1.59, 1.76) | 1.36 (1.29, 1.43) | 1.31 (1.25, 1.38) |
| No disability | 51.0 | Referent | Referent | Referent | Referent |
|  |  |  |  |  |  |
| **Hospital admissions** |  |  |  |  |  |
| Disability and ODSP | 88.3 | 5.18 (4.77, 5.63) | 4.89 (4.49, 5.34) | 2.01 (1.82, 2.22) | 1.65 (1.48, 1.84) |
| Disability and no ODSP | 27.8 | 1.64 (1.51, 1.77) | 1.88 (1.73, 2.04) | 1.46 (1.34, 1.59) | 1.38 (1.27, 1.50) |
| No disability | 17.0 | Referent | Referent | Referent | Referent |
|  |  |  |  |  |  |
| **Palliative care visits** |  |  |  |  |  |
| Disability and ODSP | 9.2 | 4.30 (3.36, 5.50) | -- | -- | -- |
| Disability and no ODSP | 2.2 | 1.01 (0.78, 1.31) | -- | -- | -- |
| No disability | 2.1 | Referent | Referent | Referent | Referent |

-- Model not estimable.

* Adjusted for socio-demographics (age; sex; neighbourhood income, households and dwellings, material resources, age and the labour force, and racialized and newcomer populations quintiles; rurality).

† Adjusts for socio-demographics and comorbidities recorded in the 5 years before the index admission.

‡ Adjusts for socio-demographics, comorbidities, and prior health service use (number of ambulatory care visits, emergency department visits, and hospital admissions; receipt of palliative care; and number of prescription drugs in the 12 months before the index admission.

**eTable 7. Cumulative effects on the disability-health service use association of adjusting sequentially for blocks of risk factors, by Ontario Disability Support Program status.**

| **Outcomes** | **Disability status** | **Proportion change of association that could be explained by collective adjustment of sequential covariate sets** | | | **Proportion change of association that could be explained by sequential covariate set adjustments** | |
| --- | --- | --- | --- | --- | --- | --- |
|  |  | **Sociodemographic  (Model 1)** | **Model 1 + Comorbidities  (Model 2)** | **Model 2 + Prior health service use  (Model 3)** | **Comorbidities  (Model 1 vs. 2)** | **Prior health**  **service use (Model 2 vs. 3)** |
| Ambulatory care visits | Disability and ODSP | 6.2 (2.7, 10.2) | 70.6 (61.2, 82.9) | 86.0 (73.8, 100.0) | 68.6 (58.9, 81.5) | 52.4 (16.2, 100.0) |
|  | Disability and no ODSP | 11.7 (8.0, 16.3) | 46.1 (40.2, 53.4) | 69.4 (62.3, 78.3) | 39.0 (33.7, 45.8) | 43.3 (34.3, 55.3) |
| Diagnostic tests | Disability and ODSP | 14.5 (10.5, 19.0) | 78.3 (67.8, 93.4) | 90.9 (77.3, 100.0) | 74.6 (62.8, 92.0) | 58.2 (19.5, 100.0) |
|  | Disability and no ODSP | 25.1 (19.6, 31.7) | 58.0 (48.6, 68.5) | 66.0 (56.2, 77.6) | 43.9 (34.5, 55.3) | 18.9 (10.4, 33.7) |
| Emergency department visits | Disability and ODSP | 6.0 (1.5, 10.7) | 55.2 (45.6, 67.4) | 68.4 (59.5, 81.4) | 52.3 (42.8, 64.0) | 29.5 (14.6, 54.9) |
|  | Disability and no ODSP | 40.4 (23.9, 67.8) | 16.9 (1.8, 36.4) | 26.1 (9.1, 50.6) | 40.8 (29.7, 56.3) | 11.1 (0.6, 39.9) |
| Hospital admissions | Disability and ODSP | 3.5 (0.5, 7.4) | 57.5 (49.8, 67.2) | 69.7 (61.2, 79.8) | 55.9 (48.3, 65.4) | 28.7 (11.8, 46.0) |
|  | Disability and no ODSP | 28.1 (16.9, 43.6) | 22.7 (8.5, 42.2) | 34.6 (19.1, 54.5) | 39.6 (29.5, 53.9) | 15.4 (2.0, 33.9) |
| Palliative care visits | Disability and ODSP | 26.0 (9.0, 67.1) | 56.9 (13.4, 100.0) | 84.9 (31.7, 100.0) | 41.8 (2.4, 100.0) | 65.0 (3.0, 100.0) |
|  | Disability and no ODSP | 100.0 (21.7, 100.0) | 100.0 (27.2, 100.0) | 100.0 (14.5, 100.0) | 47.3 (3.5, 100.0) | 14.6 (1.2, 100.0) |

**eTable 8. Rates of ambulatory care visits, diagnostic tests, emergency department visits, hospitalizations, and palliative care visits, comparing patients with and without pre-existing disabilities following hospitalization for COVID-19, using the alternative definition of disability**.**

|  | **Rate per 1000 person-months** | **RR**  **(95% CI)** | **Adjusted RR**  **(95% CI)** * | **Adjusted RR**  **(95% CI)** † | **Adjusted RR**  **(95% CI)** **‡** |
| --- | --- | --- | --- | --- | --- |
| **Ambulatory care visits** |  |  |  |  |  |
| Disability | 1468.5 | 1.47 (1.46, 1.48) | 1.40 (1.39, 1.41) | 1.18 (1.17, 1.19) | 1.08 (1.07, 1.09) |
| No disability | 999.9 | Referent | Referent | Referent | Referent |
|  |  |  |  |  |  |
| **Diagnostic tests** |  |  |  |  |  |
| Disability | 401.5 | 1.76 (1.74, 1.79) | 1.55 (1.52, 1.57) | 1.19 (1.17, 1.21) | 1.11 (1.09, 1.13) |
| No disability | 227.8 | Referent | Referent | Referent | Referent |
|  |  |  |  |  |  |
| **Emergency department visits** |  |  |  |  |  |
| Disability | 109.4 | 1.87 (1.82, 1.92) | 2.20 (2.14, 2.27) | 1.50 (1.45, 1.55) | 1.31 (1.27, 1.36) |
| No disability | 58.5 | Referent | Referent | Referent | Referent |
|  |  |  |  |  |  |
| **Hospital admissions** |  |  |  |  |  |
| Disability | 63.9 | 2.46 (2.36, 2.56) | 2.21 (2.11, 2.31) | 1.47 (1.40, 1.54) | 1.29 (1.23, 1.36) |
| No disability | 26.0 | Referent | Referent | Referent | Referent |
|  |  |  |  |  |  |
| **Palliative care visits** |  |  |  |  |  |
| Disability | 15.83 | 3.56 (3.24, 3.91) | 1.21 (1.09, 1.34) | 0.86 (0.77, 0.96) | 0.80 (0.71, 0.89) |
| No disability | 4.44 | Referent | Referent | Referent | Referent |

Definition of disability does not include osteoarthritis, glaucoma, or cataracts.

* Adjusted for socio-demographics (age; sex; neighbourhood income, households and dwellings, material resources, age and the labour force, and racialized and newcomer populations quintiles; rurality).

† Adjusts for socio-demographics and comorbidities recorded in the 5 years before the index admission.

‡ Adjusts for socio-demographics, comorbidities, and prior health service use (number of ambulatory care visits, emergency department visits, and hospital admissions; receipt of palliative care; and number of prescription drugs in the 12 months before the index admission.
